# Environmental modifiers of RTS,S/AS01 malaria vaccine efficacy in Lilongwe, Malawi

**DOI:** 10.1186/s12889-020-09039-z

**Published:** 2020-06-12

**Authors:** Griffin J. Bell, Matthew S. Loop, Tisungane Mvalo, Jonathan J. Juliano, Innocent Mofolo, Portia Kamthunzi, Gerald Tegha, Marc Lievens, Jeffrey Bailey, Michael Emch, Irving Hoffman

**Affiliations:** 1 Department of Epidemiology, Gillings School of Global Public Health, University of North Carolina, Chapel Hill, NC 27599 USA; 2 Department of Biostatistics, Gillings School of Global Public Health, University of North Carolina, Chapel Hill, NC 27599 USA; 3 University of North Carolina, Chapel Hill, NC 27599 USA; 4 University of North Carolina Project Malawi, Lilongwe, Malawi; 5 Division of Infectious Diseases, School of Medicine, University of North Carolina, Chapel Hill, NC 27599 USA; 6 GlaxoSmithKline (GSK), Wavre, Belgium; 7 Department of Pathology and Laboratory Medicine, Brown University, Providence, RI 02912 USA

**Keywords:** Malaria, Vaccine, Africa, Spatial analysis, Vaccine trial, Malawi, RTS,S/AS01, Hansen Forest cover

## Abstract

**Background:**

RTS,S/AS01 is the first vaccine against malaria to undergo pilot implementation, beginning in 2019 and vaccinating 360,000 children per year in Malawi, Ghana, and Kenya. The four-dose vaccine is given as a primary three-dose series with a fourth dose given approximately 18 months later. The efficacy of RTS,S/AS01 was variable among the 11 sites participating in the 2009–2014 phase III trial (MALARIA-055, NCT00866619), possibly due to differences in transmission intensity. However, a within-site examination of environmental factors related to transmission intensity and their impact on vaccine efficacy has yet to be conducted.

**Methods:**

We implemented the phase III RTS,S/AS01 trial at the Malawi site, which enrolled 1578 infants (6–12 weeks) and children (5–17 months) living in the Lilongwe District in Central Malawi and followed them for 3 years between 2009 and 2014. A global positioning system survey and an ecological questionnaire were conducted to collect participant household locations and characteristics, while additional data on background malaria prevalence were obtained from a concurrent Malaria Transmission Intensity (MTI) survey. Negative binomial regression models were used to assess whether the efficacy of the vaccine varied by estimated background malaria prevalence, household roof type, or amount of nearby vegetation.

**Results:**

Vaccine efficacy did not significantly vary by estimated malaria prevalence or by roof type. However, increased vegetation cover was associated with an increase in the efficacy of the three-dose primary RTS,S/AS01 series in the 18 months before the fourth dose and a decrease in the efficacy of the primary vaccine series in the second 18 months following, if the fourth dose was not given. Vegetation cover did not alter the efficacy of the fourth dose in a statistically or practically significant manner.

**Conclusions:**

Vegetation coverage in this study site might be a proxy for nearness to rivers or branching, shallow wetlands called “dambos” which could serve as breeding sites for mosquitoes. We observed statistically significant modification of the efficacy of RTS,S/AS01 by forest cover, suggesting that initial vaccine efficacy and the importance of the fourth dose varies based on ecological context.

**Trial registration:**

Efficacy of GSK Biologicals’ Candidate Malaria Vaccine (257049) Against Malaria Disease Caused by *P. falciparum* Infection in Infants and Children in Africa. NCT00866619 prospectively registered 20 March 2009.

## Background

In 2018, there were an estimated 219 million clinical malaria cases and 405,000 malaria-related deaths worldwide [1]. Although malaria is not unique to the African continent, 93% of global cases and deaths occurred there in 2018. The number of deaths in Africa due to malaria has decreased by 29% since 2010, but progress against malaria has stalled in recent years. Since 2015, estimated yearly malaria cases have increased by 7 million (~ 3%) and estimated yearly deaths have decreased by 24,000 (~ 6%) [1].

RTS,S, administered with AS01, is the only vaccine against malaria which is presently approved for use and is undergoing pilot implementation. AS01 is an Adjuvant System containing MPL, QS-21 and liposome. RTS,S/AS01 is given in four doses, consisting of three doses, administered in one-month intervals at baseline, and an additional fourth dose recommended to maximize efficacy 18 months after the third dose. The four-dose regimen of RTS,S/AS01 has been incorporated into the vaccine schedule and is being administered to 360,000 children per year in Malawi, Ghana, and Kenya as part of a pilot implementation program which began in 2019. Young children are being targeted for vaccination as malaria tends to be more severe in children under 5 years old. Children under five account for two out of every three malaria deaths worldwide [1].

The efficacy of the RTS,S/AS01 vaccine, defined as one minus the incidence rate ratio (1-IRR), was not uniform across phase III trial sites, ranging from 22% in Manhiça, Mozambique to 74.6% in Kilifi, Kenya in the 4-dose, 5–17 month-old group [2]. This between-site variation in vaccine efficacy suggests that there may be environmental factors influencing trial results. A trend has been noted: Olotu et al. showed that the vaccine tended to have a higher initial efficacy in higher transmission intensity areas, but that the efficacy waned quicker, in an extended phase II trial [3]. Tinto et al. observed negative efficacy point estimates in the last three of seven years of the phase III trial in Nanoro, Burkina Faso and Kombewa, Kenya, but not in the site with the lowest incidence of malaria: Korogwe, Kenya [4]. Both Olotu et al. and Tinto et al. suggest that the efficacy of the vaccine, in higher transmission intensity areas, could wane to less than 0. An efficacy less than 0 means that the treatment has become harmful (though the overall effect of treatment throughout the course of follow-up might still be beneficial). Whether the efficacy of RTS,S/AS01 varies in different transmission settings was identified as a critical question to address before widescale implementation of the vaccine [5].

Lilongwe, the capital city of Malawi, was a site in the phase III trial of RTS,S/AS01 and is a peri-urban area, with varying degrees of urban and rural qualities across the study site. This makes Lilongwe an ideal study area to examine within-site vaccine efficacy variation and the relationship with transmission intensity.

The objective of this analysis was to evaluate whether neighborhood- or household-level malaria risk modified the efficacy of RTS,S/AS01. First, estimated background malaria prevalence within the Lilongwe site was considered as a potential modifier. Second, as malaria risk may have varied from household to household, we considered household materials (roof type) and immediate micro-environment (nearby vegetation) as potential modifiers.

## Methods

### Study population and design

The methods and the main results of the phase III clinical trial, from which our data are derived, have been reported according to CONSORT guidelines [2]. Our study population included infants aged 6–12 weeks and children aged 5–17 months (*n* = 1578) enrolled in MALARIA-055 (NCT00866619), which was the 2009–2014 phase III trial of RTS,S/AS01 (GSK) in Lilongwe, Malawi. The analysis population included only children aged 5–17 months (*n* = 783), since infants will not be given the vaccine moving forward due to low efficacy [2]. MALARIA-055 was an individually randomized, double-blind trial. Participants were stratified based on age group, infant (6–12 weeks) or child (5–17 months), and then randomized into three-dose control/control dose (C3 + C), three-dose vaccine/control dose (R3 + C), and three-dose vaccine/fourth dose (R3 + R) groups. All participants received 3 doses of the RTS,S/AS01 vaccine (R3) or a control vaccine (C3) at baseline and received a fourth (+R) or control dose (+C) after 18 months follow up. We administered an ecological survey up to 4 separate times during a participant’s enrollment in the trial, targeting months 6, 12, 18 and 24 of enrollment. This survey measured bed net use as well household building materials. Bed nets were given to each trial participant upon enrollment, by design [2], and the proportion using a net in this population was over 95%, as measured by the ecological survey.

### Outcome

Cross-sectional screening for Malaria was performed on months 20 and 32. Otherwise, Malaria cases were detected throughout the trial by passive case detection, i.e. if a participant presented with a current or recent fever. If a participant was found to have malaria, they received treatment in accordance with national guidelines [2]. For the purpose of this analysis, an episode of clinical malaria was defined as illness accompanied by an axillary temperature of at least 37.5 °C and presence of *Plasmodium falciparum* asexual parasitemia (> 5000 parasites per mm^3^) measured using microscopy [2].

### Potential modifiers of vaccine efficacy

Estimates of background malaria prevalence were derived from a 2011–2013 malaria transmission intensity (MTI) study. The study conducted an annual cross-sectional survey of *P. falciparum* prevalence administered concurrently with the phase III RTS,S/AS01 trial in each study site. The survey was implemented during the peak malaria season (February–June) and enrolled 400 under-five-year-olds annually, from the same catchment area of the phase III trial with enrollment in the phase III trial being an exclusion criterion [6]. Of the 1200 under-five-year-olds recruited from the three MTI surveys, 1183 (98.6%) had geographic information and a malaria test and were used to construct a raster of estimated malaria prevalences throughout Lilongwe using a thin plate regression spline (TPRS). A TPRS is a 2-dimensional spline which includes a penalty for ‘wigglyness’ (the squared second derivative) in the surface [7]. The two dimensions, in the spatial case, are longitude and latitude. Fitted malaria prevalence values from the TPRS were extracted to the household point locations of phase III trial participants.

Household roof type was considered as potential modifier of vaccine efficacy, as modern housing can act as a physical barrier against mosquitoes, reducing the risk of malaria [8]. It can also act as a proxy measurement for socio-economic status: Household construction materials are frequently used to determine wealth, notably in Demographic and Health Surveys (DHS) [9]. Participants almost always had grass or metal sheet roofs, and almost all participants with grass roofs had mud walls and they were much less likely to have glass or screened windows.

**Fig. 1:**
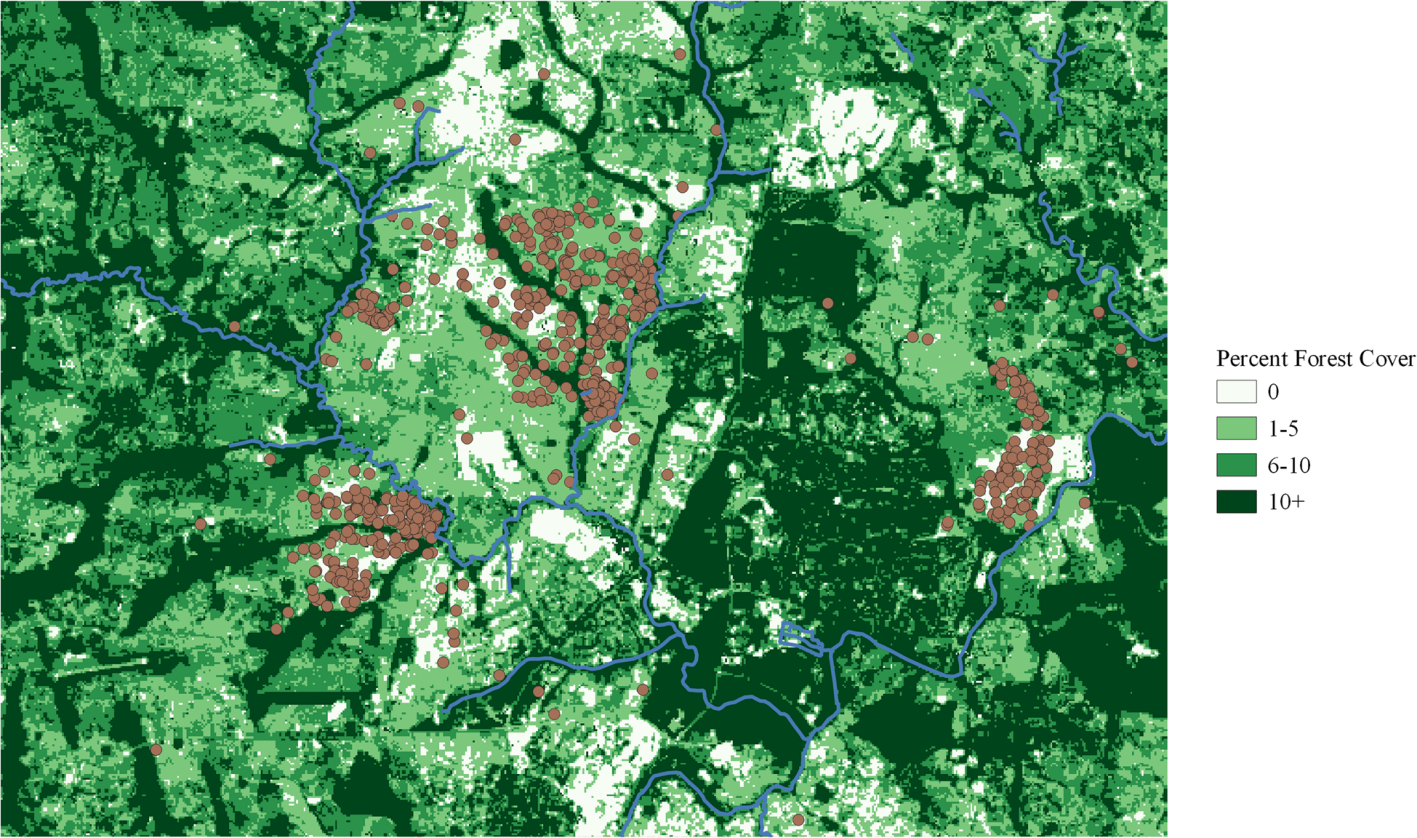
Forest/vegetation cover in Lilongwe City overlaid with OpenStreetMap rivers and participant household point locations *This map was created in QGIS version 3.4.7 using Hansen forest cover data (available at:**https://earthenginepartners.appspot.com/science-2013-global-forest/download_v1.6.html**)*

When considering high-risk microenvironments for malaria transmission, it is critical to consider standing water, which serves as a breeding ground for anopheline mosquitoes and ties malaria risk to land cover [10]. River and lake data will not fully capture the geospatial distribution of standing water, as water can accumulate as the result of rainfall or be maintained in shallow standing water sources. In Malawi, one such standing water source is the dambo, a shallow body with a branching river-like form [11]. Hansen forest cover data, a 30-m resolution satellite raster product measuring year-round forest/vegetation coverage in the form of percent canopy closure for all vegetation taller than 5 m in height, picked up branching river-like patterns of vegetation which were not considered to be rivers or streams in OpenStreetMap data (Fig. 1) [12, 13]. Vegetation coverage is measured as the percent of coverage attributable to vegetation, as previously defined, in each grid cell. We used this year-round vegetation coverage as a proxy for suitable environmental conditions (i.e. standing water) for anopheline mosquitoes. Using the Hansen forest cover raster for the year 2000 (available at: https://earthenginepartners.appspot.com/science-2013-global-forest/download_v1.6.html), we considered (percent) vegetation cover within 100 m of each household. The buffer of 100 m was chosen in order to consider values outside the immediate grid cell of each household but to also maintain a microenvironment for that household. As a sensitivity analysis, we also considered the value at the point location of the household and the average value within 200 m.

All maps and figures were created in R version 3.5.1, except for Fig. 1, which was created in QGIS version 3.4.7 [14].

### Statistical analysis

Between the levels of our potential modifiers, we compared efficacies (1-IRR) of the primary RTS,S/AS01 vaccine series in the first 18 months, the primary RTS,S/AS01 vaccine series in the second 18 months (without the fourth dose), and the fourth dose in the second 18 months. First, we investigated whether the prevalence of malaria in under-fives in the study area, obtained from the MTI survey, modified the effect of the vaccine on the multiplicative scale. Then, this was repeated for roof type and vegetation coverage.

We fit generalized mixed effects negative binomial models, with a log link function, in R version 3.5.1 using the “lme4” package [14, 15]. In our models, subscript “*i*” corresponds to participant *i* and subscript “*j*” corresponds to time period *j*. We used a random intercept for each participant (*b*_*i*_) to account for within-subject correlation due to repeated measures, and a time (days) offset, *T*_*ij*_. *Y*_*ij*_ is the number of malaria cases experienced by participant *i*, during period *j*.

The R3 + C and R3 + R groups receive the same treatment until the fourth dose is given at 18 months. Thus, the model must allow for these groups to have the same treatment effect in the first 18 months yet different treatment effects in the second 18 months. The model accounts for this by including an [(*A*_*i*_ + *B*_*i*_) ∗ (1 − *P*_*ij*_)] term, where *A*_*i*_ is one if participant *i* received the initial 3 doses of RTS,S/AS01 at baseline and a control vaccine at 18 months (R3 + C) and zero otherwise and *B*_*i*_ is one if participant *i* received the initial 3 doses of RTS,S/AS01 at baseline and the fourth dose at 18 months and zero otherwise (R3 + R). *A*_*i*_ and *B*_*i*_ will both be zero if the participant received the control vaccine at baseline (C3 + C). *P*_*ij*_ is zero if period *j* occurred during the first 18 months of follow-up and one if period *j* occurred during the second 18 months. Thus, the [(*A*_*i*_ + *B*_*i*_) ∗ (1 − *P*_*ij*_)] term evaluates to 1 if the participant is in either the R3 + C or the R3 + R group and the time being considered is in the first 18 months, and will be 0 otherwise. Similarly, (*A*_*i*_ ∗ *P*_*ij*_) and (*B*_*i*_ ∗ *P*_*ij*_) terms are included to allow for separate effects of the R3 + C and R3 + R groups, but only in the second 18 months. Therefore, the general form of our model of clinical malaria cases is:

$$ \mathit{\log}\left(E\left[{Y}_{ij}\right]\right)=\alpha +{b}_i+{\beta}_1\left[\left({A}_i+{B}_i\right)\ast \left(1-{P}_{ij}\right)\right]+{\beta}_2{P}_{ij}+{\beta}_3\left({A}_i\ast {P}_{ij}\right)+{\beta}_4\left({B}_i\ast {P}_{ij}\right)+\mathit{\log}\left({T}_{ij}\right),{b}_i\sim N\left(0,{\sigma}^2\right). $$

From this model, three specific efficacies (1-IRR) were calculated: 1) the primary vaccine series in the first 18 months, 2) the primary vaccine series in the second 18 months (without receiving a fourth dose), and 3) the fourth dose in the second 18 months. For each specific efficacy, we will determine whether our ecological variables are modifiers on the multiplicative scale by including interaction terms between the corresponding treatment term in question and the ecological variable that might influence efficacy. For each ecological variable, one model will include interactions for efficacies in the first 18 months (the three-dose primary vaccine series), and another model will include interactions for efficacies in the second 18 months (the three-dose primary vaccine series and the fourth dose).

## Results

### Study population

**Fig. 2:**
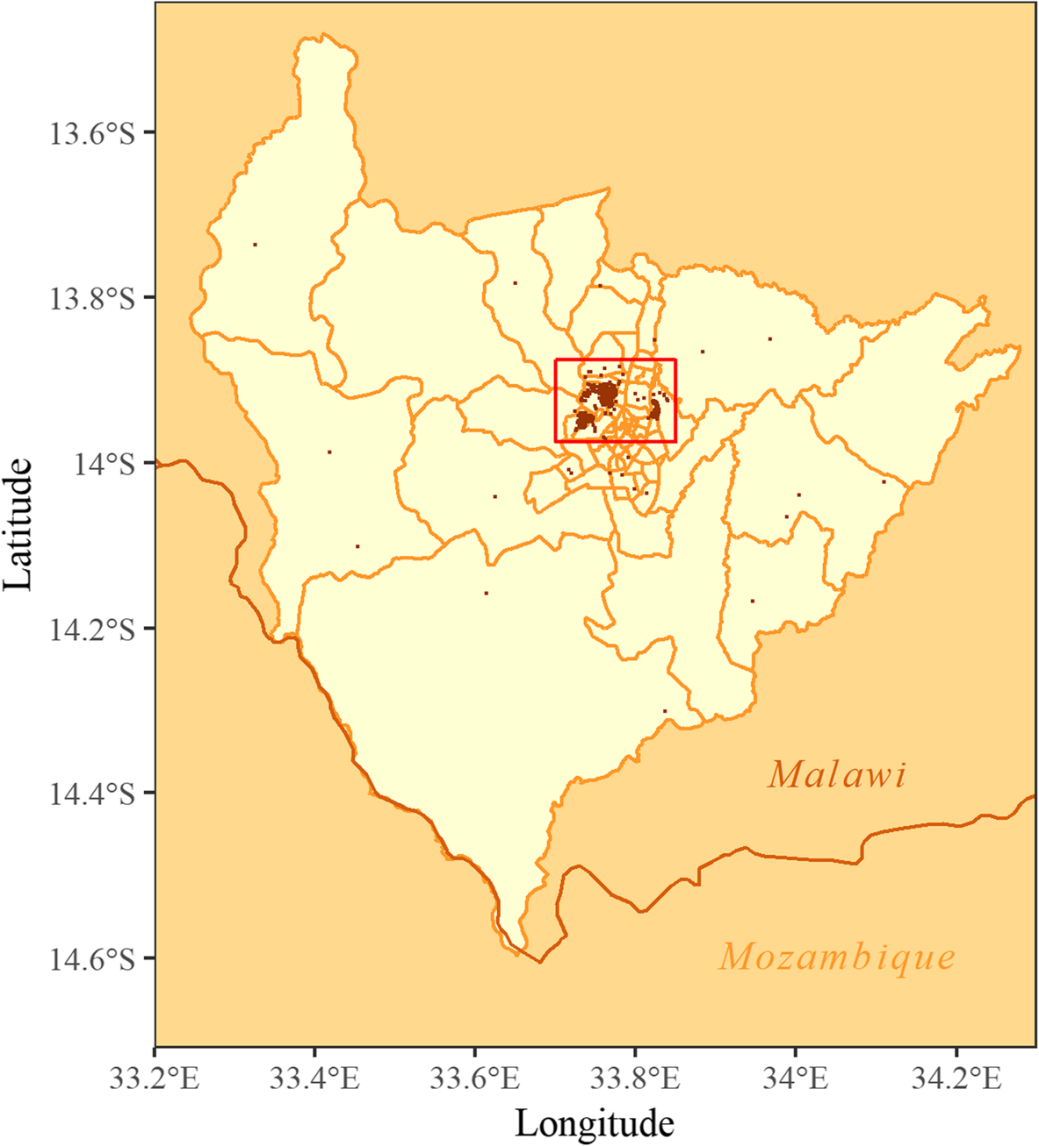
Household locations of participants in MALARIA-055 in Lilongwe District, plotted over traditional authority boundaries. *This map was created in R version 3.5.1*

Data for 783 children were available, yet 26 participants (3.32%) did not have vaccination information or had zero days of follow-up and were immediately excluded, leaving 757 children (96.68%). Household locations for these participants within Lilongwe district are displayed in Figs. 1 and 2. The region in the red box in Fig. 2 corresponds to the region displayed in Fig. 1 and subsequent maps.

### Summary statistics

A description of the 757 participants with treatment data and non-zero follow-up time is displayed in Table 1. In the ecological survey, 64 (8.45%) of the 757 children were missing data on roof type. 27 (3.57%) participants were missing vegetation cover data due to a geolocation that was missing or outside Lilongwe district and 49 (6.47%) participants were missing MTI prevalence due to not being within the smoothing estimation range. When a participant was missing a variable needed for a specific model, they were excluded for that model (complete case analysis). There were no important differences between treatment groups in terms of the distribution of gender or ecological variables, as would be expected in a randomized trial. There was a difference between treatment groups in terms of malaria incidence.

**Table 1:** Summary of participants by treatment group

***Variable***	***C3 + C*** ***(n = 248)***	***R3 + C*** ***(n = 262)***	***R3 + R*** ***(n = 247)***	***All*** ***(n = 757)***
***Gender N (%)***				
*Male*	130 (52.42)	127 (48.47)	121 (48.99)	378 (49.93)
*Female*	117 (47.18)	135 (51.53)	125 (50.61)	377 (49.80)
*Missing*	1 (00.40)	0 (00.00)	1 (00.40)	2 (00.26)
***First Measured Roof Type N (%)***				
*Grass*	49 (19.76)	65 (24.81)	48 (19.43)	162 (21.40)
*Metal*	177 (71.37)	176 (67.94)	178 (72.06)	531 (70.15)
*Missing*	22 (08.87)	21 (8.02)	21 (08.50)	64 (08.45)
***Percent Vegetation Cover (100 m) N (%)***				
*Less than 1%*	71 (28.63)	72 (27.48)	70 (28.34)	213 (28.14)
*1 to 5%*	120 (48.39)	138 (52.67)	126 (51.01)	384 (50.73)
*5 to 10%*	41 (16.53)	40 (15.27)	39 (15.79)	120 (15.85)
*Over 10%*	5 (02.02)	5 (01.91)	3 (01.21)	13 (01.72)
*Missing*	11 (04.44)	7 (02.67)	9 (03.64)	27 (03.57)
***Estimated MTI Prevalence N (%)***				
*Less than 1%*	32 (12.90)	26 (09.92)	25 (10.12)	83 (10.96)
*1 to 5%*	115 (46.37)	122 (46.56)	110 (44.53)	347 (45.84)
*5 to 10%*	55 (22.18)	57 (21.76)	54 (21.86)	166 (21.93)
*Over 10%*	31 (12.50)	39 (14.89)	42 (17.00)	112 (14.80)
*Missing*	15 (06.05)	18 (06.87)	16 (06.48)	49 (06.47)
***Incidence Rate (Cases per Year)***				
*First 18 Months*	0.50	0.28	0.22	0.33
*Second 18 Months*	0.32	0.26	0.17	0.25
*All Three Years*	0.42	0.28	0.19	0.30

### Estimated MTI prevalence and vaccine efficacy

**Fig. 3:**
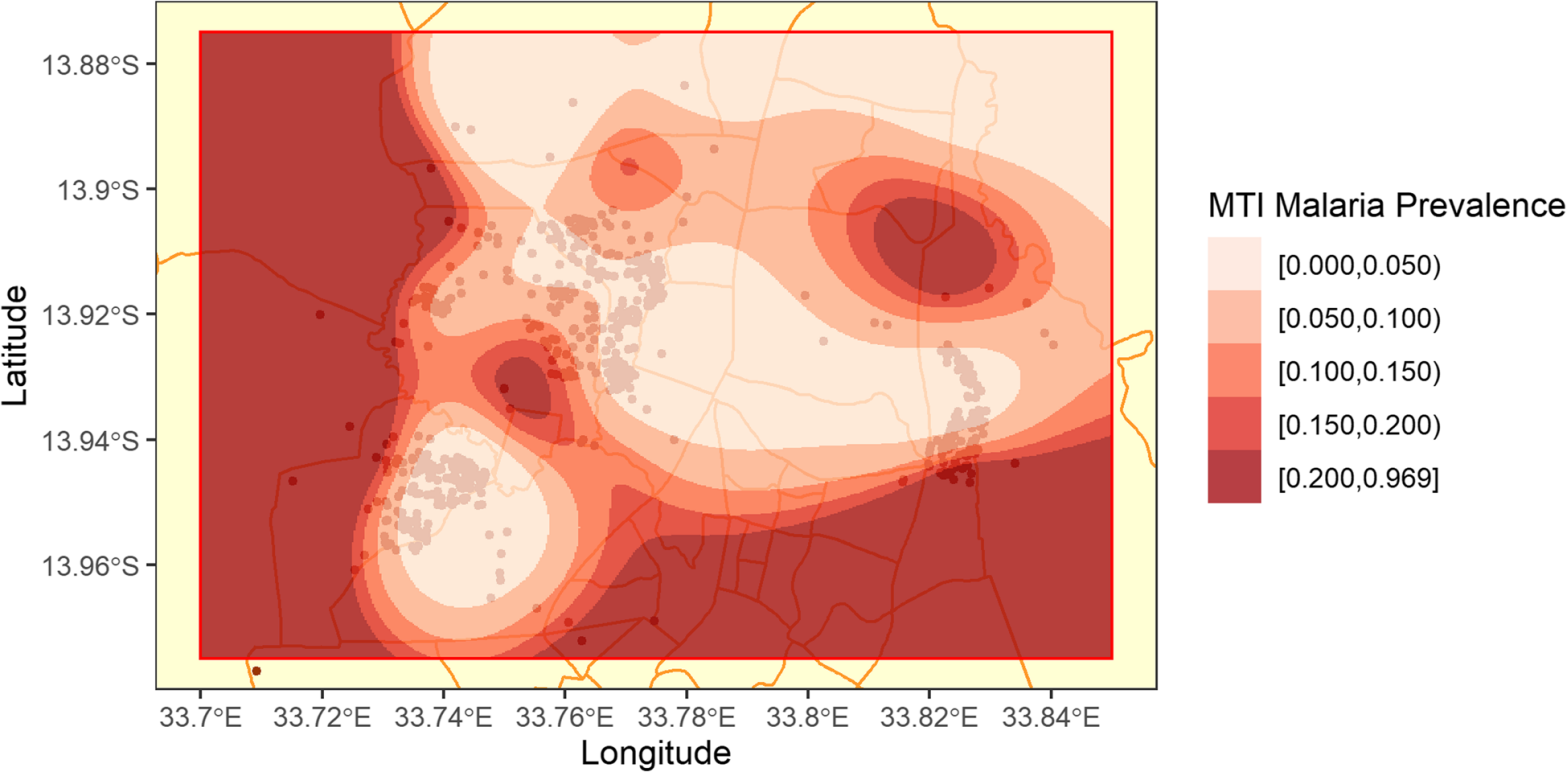
Estimates of malaria prevalence in under-fives in Lilongwe city from 2011 to 2013 according to the MTI survey data. *This map was created in R version 3.5.1*

Estimates of malaria prevalence in Lilongwe city during peak malaria season, according to the three Malaria Transmission Intensity surveys done each year from 2011 to 2013, are shown in Fig. 3. Estimated average under-five malaria prevalence, extracted from observed participant household locations, varied between 0.50 and 66.44%, with most participants residing in relatively lower prevalence areas. A model with an interaction term between smoothed MTI prevalence at the household point and vaccination status was constructed, but estimated differences in efficacy due to this prevalence were not statistically significant for the primary vaccine series in the first 18 months (*p* = 0.92) or the second 18 months (*p* = 0.17), and also not for the fourth dose (*p* = 0.69). MTI prevalence was significantly associated with participant risk of malaria: an increase of 1 % in MTI prevalence yields an incidence rate ratio of 1.04 (95% CI: 1.02 to 1.06).

### Roof type and vaccine efficacy

**Fig. 4:**
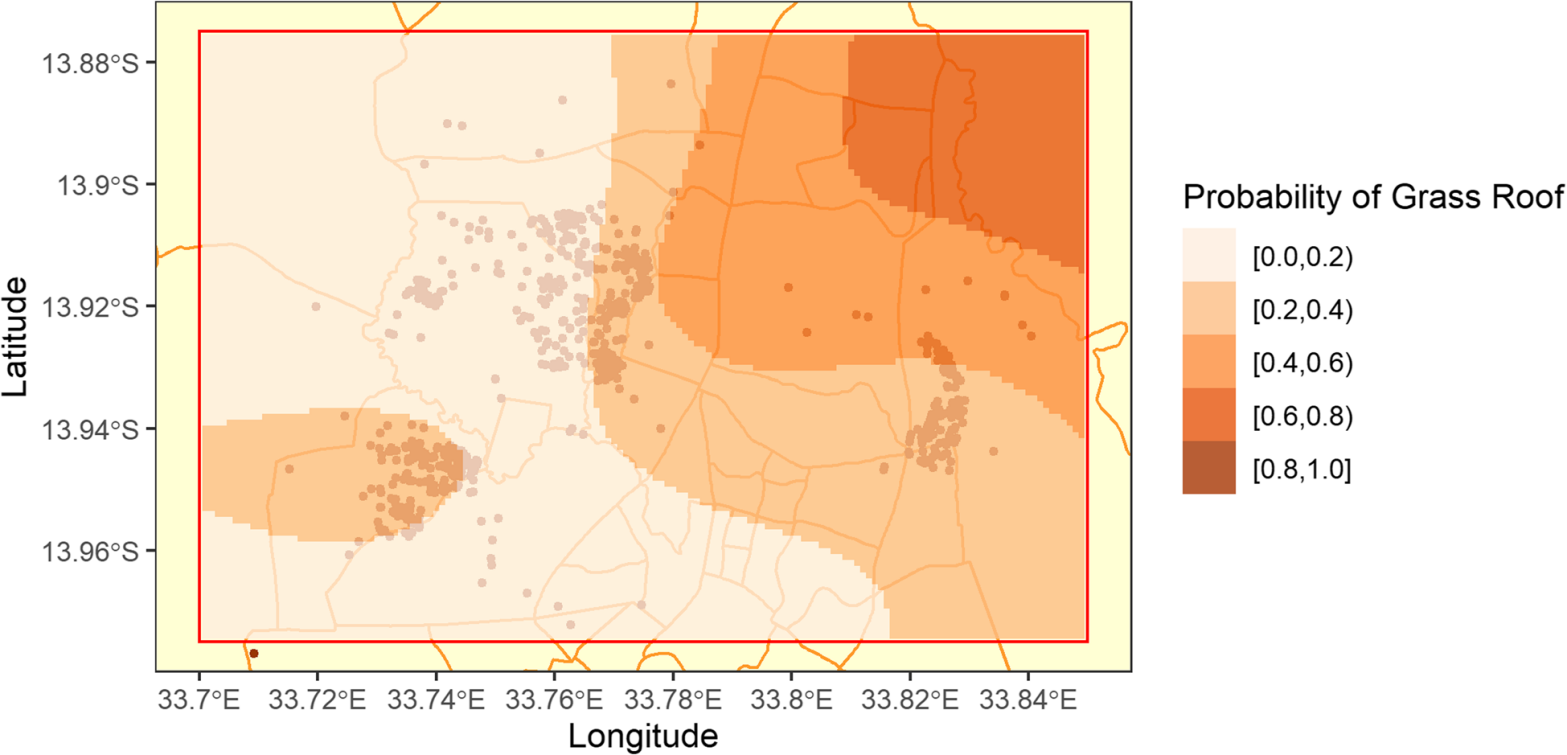
Probability of a participant having a grass roof (instead of a metal roof) throughout Lilongwe City *This map was created in R version 3.5.1*

**Fig. 5:**
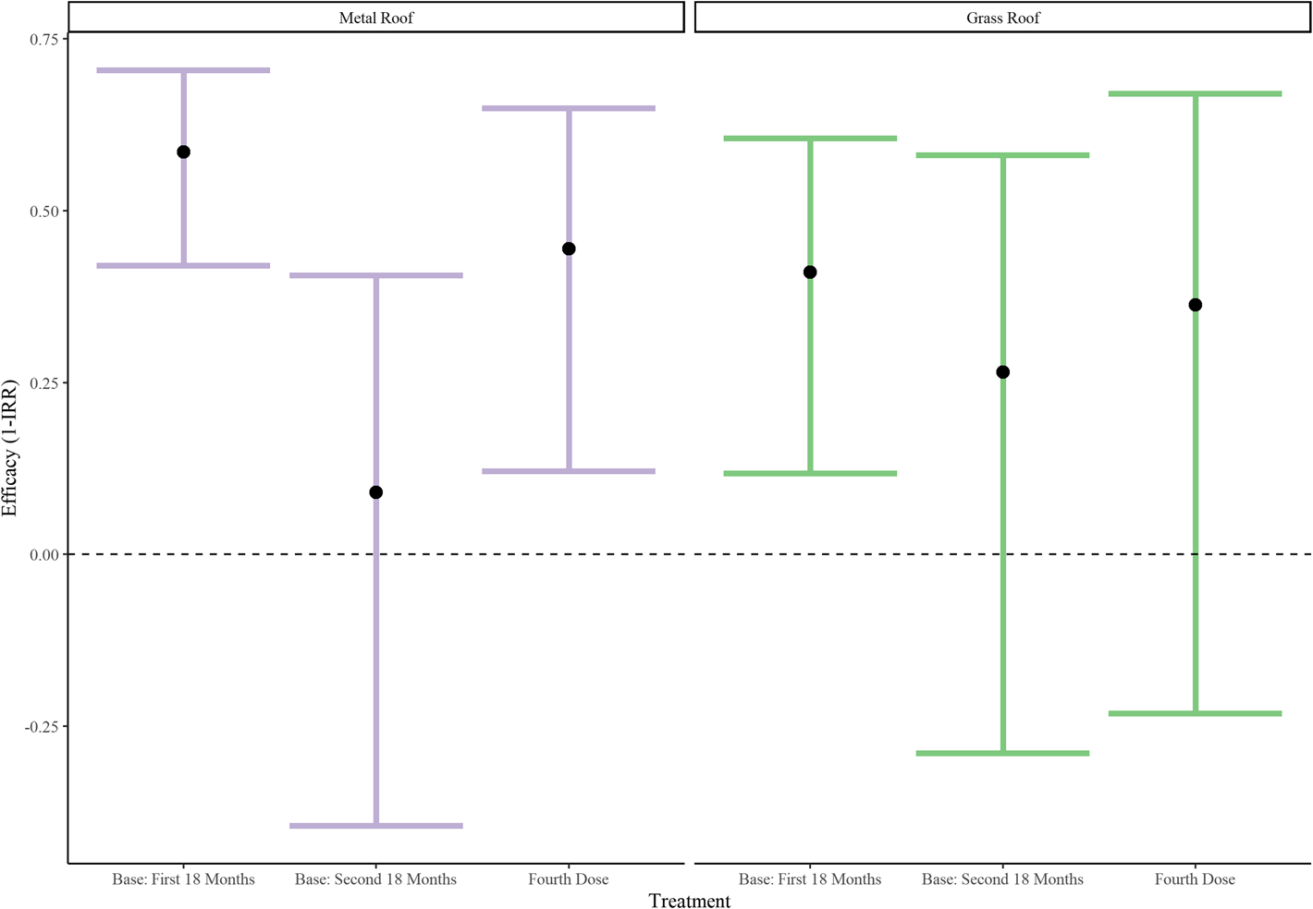
Vaccine efficacy by roof type

The estimated distribution of roofing materials throughout the study area can be seen in Fig. 4, while Fig. 5 displays the estimated vaccine efficacies for each roof type. Having a grass roof, versus metal, was positively associated with malaria incidence (IRR: 1.76, 95% CI: 1.39 to 2.22) and potentially influenced the primary vaccine series (R3) efficacy in the first 18 months (*p* = 0.09). Estimated efficacies for the first 18 months in participants with grass roofs and metal roofs were 41.05% (95% CI: 11.78 to 60.61%) and 58.59% (95% CI: 42.00 to 70.44%) respectively. However, roof type did not significantly impact the efficacy of the primary vaccine series in the second 18 months (*p* = 0.53) or the fourth dose (*p* = 0.73)

### Vegetation cover and vaccine efficacy

**Fig. 6:**
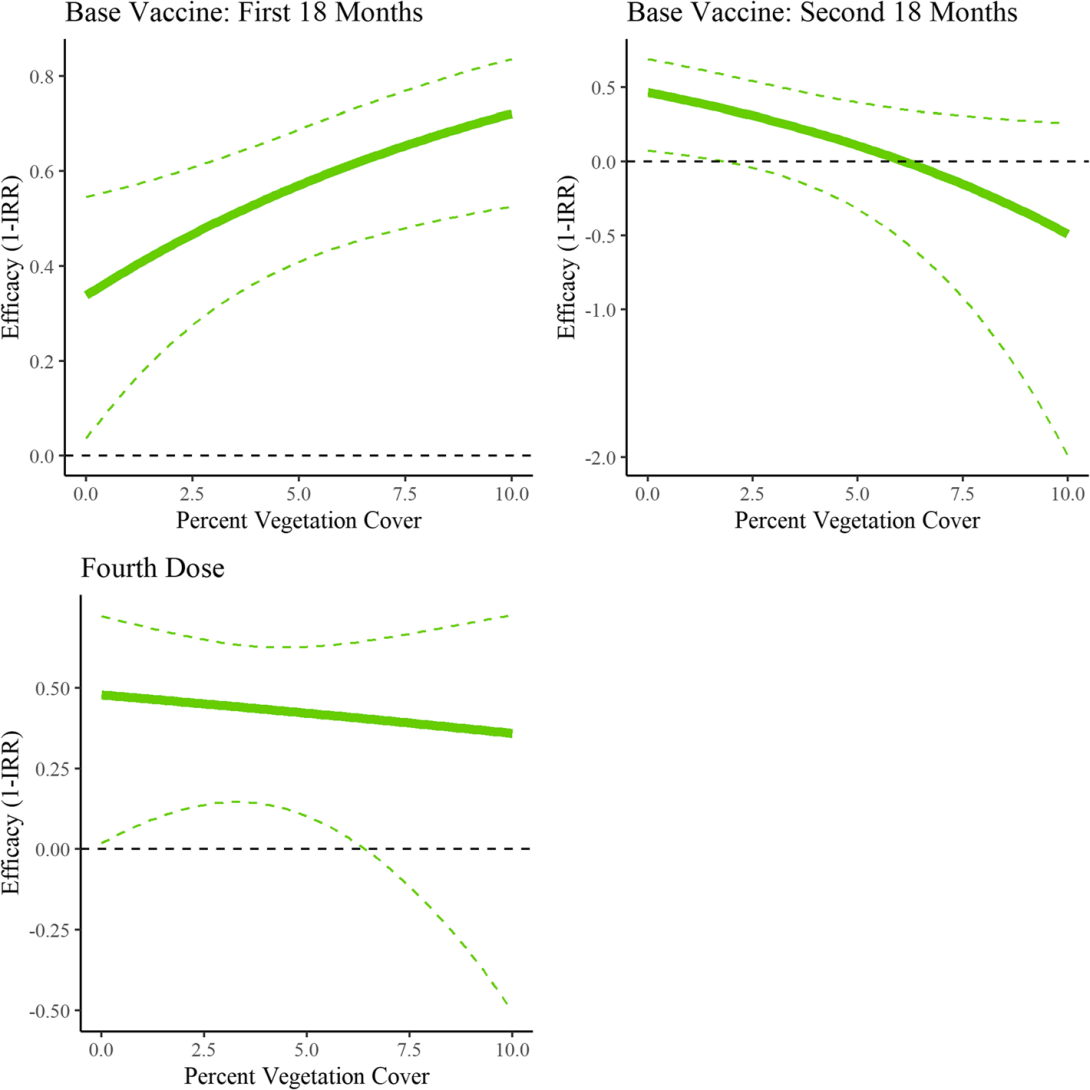
Vaccine efficacy by vegetation cover (100 m buffer)

The values of vegetation cover throughout the study area can be seen in Fig. 1. Among the households in this study, vegetation coverage within 100 m ranged from 0 to 22%, though most values occurred between 0 and 10%. Figure 6 displays the estimated vaccine efficacies between 0 and 10% vegetation cover. A 1 % increase vegetation cover was significantly associated with increased malaria incidence (IRR: 1.15, 95% CI: 1.10, 1.20), an increase in primary vaccine series efficacy in the first 18 months (*p* = 0.01), and a decrease in primary vaccine series efficacy in the second 18 months (*p* = 0.04). Increased vegetation cover was not significantly associated with fourth dose efficacy (*p* = 0.74). At 0% vegetation cover, the primary vaccine series (R3) series had an estimated 33.84% (95% CI: 3.68 to 54.56%) efficacy in the first 18 months. Without receiving a fourth dose (R3 + C), the vaccine series had an estimated 46.60% (95% CI: 7.43 to 69.19%) efficacy in the second 18 months. If a fourth dose had been received (R3 + R), the estimated efficacy was similar, at 47.76% (95% CI: 1.81 to 72.21%) efficacy in the second 18 months. At 10% vegetation cover, the primary vaccine series (R3) series had an estimated 72.11% (95% CI: 52.52 to 83.62%) efficacy in the first 18 months. Without receiving a fourth dose (R3 + C), the primary vaccine series had an estimated − 48.98% (95% CI: − 198.94 to 25.76%) efficacy in the second 18 months – the point estimate indicating a harmful effect but with values indicating small protective effects within the confidence interval. If a fourth dose had been received (R3 + R), the efficacy point estimate was improved at 35.84% (95% CI: − 50.37 to 72.62%) in the second 18 months, but with a large confidence interval and potential for harm. Patterns largely held in the sensitivity analysis, which instead of a 100-m buffer, used point extraction or a 200-m buffer (**Supplementary Figs.****1****and****2**), though the efficacy trend for the fourth dose changed depending on the buffer used.

## Discussion

The 2009–2014 phase III trial, MALARIA-055, found that RTS,S/AS01 was not equally efficacious among trial sites. One possible explanation for this result was that differing transmission rates created variability in the effect of the treatment. Lilongwe, Malawi is a peri-urban city, meaning that ecological factors, and thus transmission intensity, are likely variable throughout the site. Using household locations and micro-environmental variables as proxies for household-specific transmission intensity, we were able to test whether RTS,S/AS01 efficacy varied throughout the study site.

Though MTI prevalence was not found to significantly modify vaccine efficacy, it is possible that built and natural micro-environments better predict the household level risk of clinical malaria than prevalence smoothed over the study site. For example, our data show that a 1% increase in forest cover was associated with a greater increase in trial malaria incidence than a 1% increase in MTI background malaria prevalence (IRR 1.15 versus 1.04). Examining the interaction between roof type and the vaccine delivered little of interest, although there was potentially an increase in efficacy in the first 18 months among those with metal roofs (versus grass); the comparison was borderline significant. However, since roof type is spatially distributed, it may be that this result is representative of varying malaria risk due to differences in the natural environment.

A potentially coherent story can be drawn from the results examining vegetation cover, which served as a proxy for nearness to standing water, often in the form of branching wetlands (dambos). In the first 18 months after primary vaccination, vaccine efficacy tended to be higher in high transmission intensity localities, indicated by higher vegetation cover. However, in these high vegetation cover areas, those who received the primary vaccine series (but not the fourth dose) experienced a greater risk of malaria compared to the control group in the second 18 months, indicating harm. As suggested by Olotu et al., this may be due to the waning efficacy of RTS,S/AS01, combined with the lack of acquired immunity from natural infections, prevented by the previous higher efficacy period [3]. Those in the control group were more likely to experience infections in the first 18 months, and thus were more likely to develop naturally acquired immunity. The fourth dose is thus crucial in high transmission intensity settings, in order to protect those who received the primary vaccine series. For those in low transmission intensity localities, the fourth dose at 18 months is less urgent, as the primary vaccine series retains its efficacy relative to controls in the second 18 months. This has implications for implementation, as it can be difficult to ensure that a child returns a year and a half after primary vaccination for the fourth dose. This could be even more difficult for those residing in rural areas with lower healthcare access and where malaria incidence is often higher.

In addition to Olotu et al., other groups have noted the phenomenon of “rebound malaria” [4, 16]. Tinto et al. observed a negative efficacy point estimate in the last three of 7 years of follow-up in Nanoro, Burkina Faso for both the three- and four-dose vaccines and in Kombewa, Kenya for the three-dose vaccine only. Vaccine efficacy remained positive during this period in Korogwe, Kenya. Interestingly, Nanoro experienced the highest incidence of malaria in the control group (1.998 to 3.124 per-person-year), followed by Kombewa (1.160 to 1.418 PPY), and then Korogwe (0.165 to 0.368 PPY) [4]. Dicko and Greenwood summarized the results from Olotu et al. and Tinto et al., and suggested that “vaccinated children might need to receive additional support during the period of enhanced risk through education, improved access to treatment, and regular distribution of insecticide-treated bed nets” [16]. A previous analysis of phase III data from our group found that co-administration of the four-dose vaccine with bed nets in Lilongwe would be similarly cost-effective over 3 years compared to vaccine administration alone, and thus, coadministration of the vaccine and bed nets could be part of the solution. However, vaccine efficacy was assumed to be homogeneous within Lilongwe District in this analysis [17].

It is still not clear whether malaria incidence influences the efficacy of RTS,S/AS01. One study, also using the phase III data from Lilongwe, along with precipitation data, found that there was no association between rainfall and the efficacy of RTS,S/AS01 in Lilongwe, Malawi [18]. However, a different model was used that did not treat the R3 + C and R3 + R groups as the same in the first 18 months, and the authors accounted for waning efficacy by calculating efficacies for each year of follow up but produced only one *p*-value for the interaction between treatment and rainfall. As we observe in this study, there can be opposite impacts on efficacy in the first and second 18 months, so if the interaction term is concerning total follow-up time, these effects could be averaged out.

### Strengths and limitations

The simplest way to model this data would have been to investigate the three treatment groups (C3 + C, R3 + C, R3 + R) over a time period, and then evaluate whether any ecological variable modifies the efficacy of R3 + C or R3 + R versus C3 + C or R3 + R versus R3 + C. However, R3 + C and R3 + R had the exact same exposure for the first 18 months of the study, and treating them as different during this time needlessly decreases the sample size for comparing the R3 group against the C3 group. Additionally, comparisons of R3 + C and R3 + R will be biased towards the null if any risk time during the first 18 months is included. Furthermore, if an ecological variable had opposite effects on efficacy in the first and second 18 months, the effect could be canceled-out when considering the full period at once. One strength of our study is that our statistical model allowed us to avoid these pitfalls and directly calculate efficacies for the three-dose primary vaccine series (in the first and second 18 months) and the fourth dose, in the same model. In fact, we do uncover opposite effects on efficacy in the first and second 18 months with this method that likely would have been lost otherwise.

Another major strength of our study is the availability of household point locations and ecological characteristics of households and communities. Our study is the first to use household-level information to investigate ecological impacts on RTS,S/AS01 efficacy within a site. Using point locations allows us to avoid introducing potential biases associated with enforcing subjective aggregations.

Our study had a few limitations as well. The Hansen forest cover data is from the year 2000 and could be outdated, though the underlying forces that influenced the presence of dambos and other standing water sources likely remained comparable between measurement and the study period. Furthermore, we note that Hansen forest cover is a year-round product, meaning that seasonal variations in the forest cover were not considered. Thus, this analysis does not directly investigate the effect of the seasonal increases in forest/vegetation cover on the efficacy of RTS,S/AS01. Rather, it investigates whether residing within an area which has more average forest cover, over the course of a year, impacts the efficacy of the vaccine.

Another limitation is that malaria cases were detected mostly through passive surveillance, which has the potential to introduce bias. Finally, this study concerns only one site of the phase III trial, and therefore cannot directly investigate potential reasons for the heterogeneity of efficacy between sites.

## Conclusion

Vegetation coverage in this study site might be a proxy for nearness to rivers and branching, shallow wetlands called “dambos,” which could serve as breeding sites for mosquitoes. We observed statistically significant modification of the efficacy of RTS,S/AS01 by forest cover, suggesting that initial vaccine efficacy and the importance of the fourth dose varies based on ecological context. Further research is needed to investigate whether this heterogeneity is due to environmental variables which influence transmission intensity or other factors such as parasite or human genetics. If it is true that malaria incidence increases initial vaccine efficacy or modifies the importance or optimal timing of the fourth dose, these findings have important implications for the implementation of the vaccine. The observed increased efficacy in high forest cover areas in the first 18 months after vaccination is encouraging, as these are areas with a higher burden of malaria. However, the fourth dose seems crucial in these settings. Failure to receive the fourth dose in high forest cover areas could result in more cases of malaria in the second 18 months in vaccinated persons than in the unvaccinated persons, though the increase in cases in the second 18 months is estimated to be smaller than the number of prevented cases in the first 18 months. To address this public health concern, community education programs could stress the importance of returning for on-time administration of the fourth dose of RTS,S/AS01 in high-incidence areas.

## Supplementary information

**Additional file 1:Figure 1.** Vaccine efficacy by vegetation cover (point extraction).

**Additional file2:Figure 2.** Vaccine efficacy by vegetation cover (200 m buffer).

## Data Availability

The datasets analyzed during the current study are not publicly available due to the inclusion of participant household point locations but are available from the corresponding author on reasonable request. Hansen forest cover data be obtained online from http://earthenginepartners.appspot.com/science-2013-global-forest.

## Abbreviations

AS01: An adjuvant system containing MPL, QS-21 and liposome
MPL: 3-O-desacyl-4′-monophosphoryl lipid A
MTI: Malaria Transmission Intensity
IRR: Incidence Rate Ratio
GSK: GlaxoSmithKline
R3: Corresponding to two of the three study groups which received three doses of RTS,S at baseline.
C3: Corresponding to one of the three study groups which received three doses of a control vaccine at baseline.
+R: A booster dose of RTS,S at 18 months.
+C: A control booster at 18 months.
R3 + R: Corresponding to one of the three study groups which received three doses of RTS,S at baseline and a booster dose of RTS,S at 18 months.
R3 + C: Corresponding to one of the three study groups which received three doses of RTS,S at baseline and a control booster at 18 months.
C3 + C: Corresponding to one of the three study groups which received three doses of a control vaccine at baseline and a control booster at 18 months.
TPRS: Thin-Plate Regression Spline
DHS: Demographic and Health Surveys
