# Supplementary figures and images for: Environmental modifiers of RTS,S/AS01 malaria vaccine efficacy in Lilongwe, Malawi
Source: BMC Public Health. 2020 Jun 12;20:910. doi: 10.1186/s12889-020-09039-z (PMC7291743; doi:10.1186/s12889-020-09039-z)

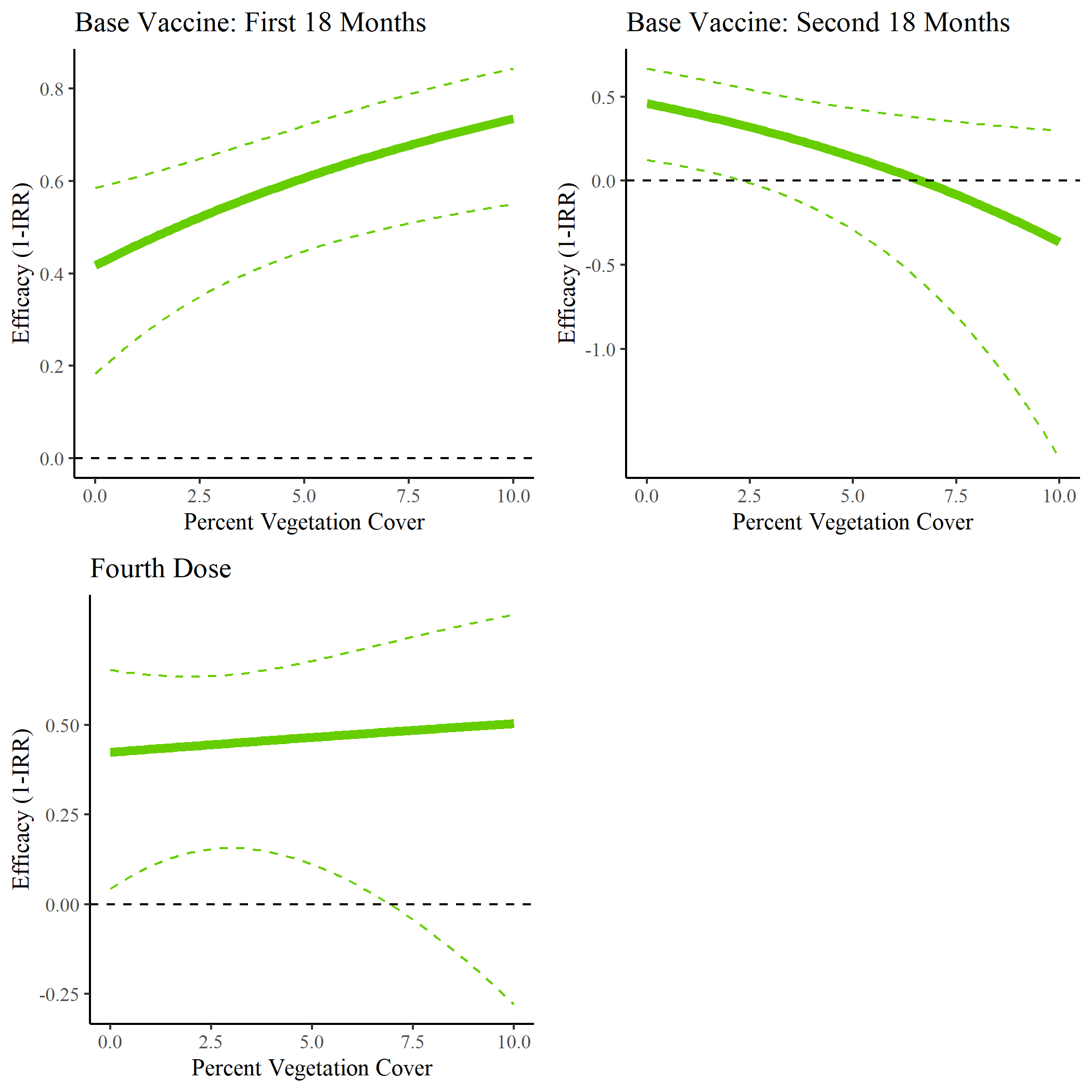

Supplement: Supplementary file 1 — Additional file 1:Figure 1. Vaccine efficacy by vegetation cover (point extraction). [file 12889_2020_9039_MOESM1_ESM.png]

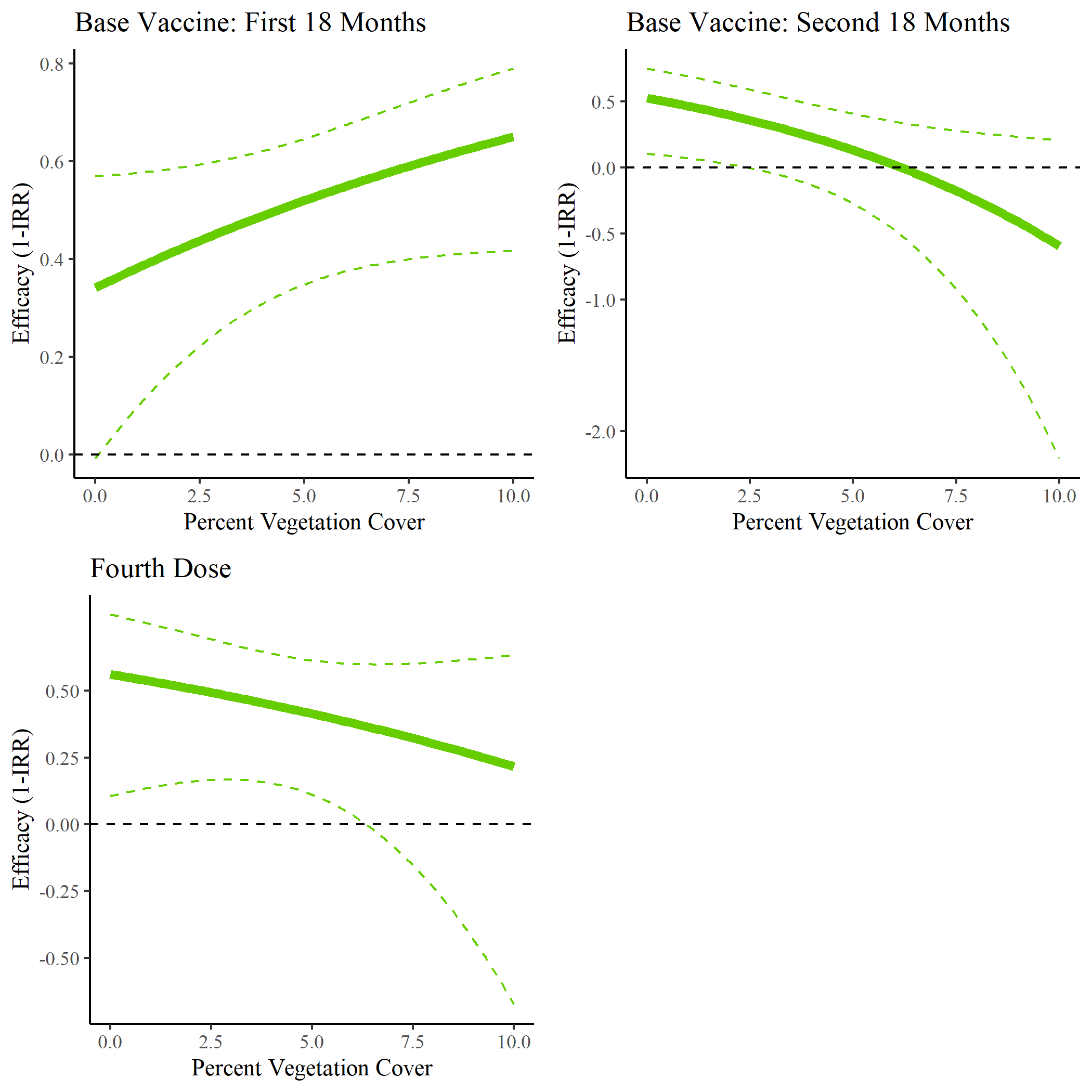

Supplement: Supplementary file 2 — Additional file2:Figure 2. Vaccine efficacy by vegetation cover (200 m buffer). [file 12889_2020_9039_MOESM2_ESM.png]
